# Review of “Brain‐Computer Interfaces, principles and practise”, edited by Jonathan R. Wolpaw and Elizabeth Winter Wolpaw

**DOI:** 10.1186/1475-925X-12-22

**Published:** 2013-03-16

**Authors:** Maureen Clerc

**Affiliations:** 1, Athena project‐team, INRIA, 06902 Sophia Antipolis, France

**Keywords:** Brain computer interfaces, Assistive technology, Translational research, Neurophysiology, Medical devices, Electroencephalography, Multiunit arrays, Electrocorticography

## Main text

The book consists of 25 chapters organized into four main parts (II to V), along with two introductory and conclusive parts. Each chapter includes an extensive list of bibliographic references, and an 8‐page index can be found at the end of the book. Its quite large dimensions make it a book to use as reference in the lab rather than to read during travel. A remarkable feature of this book is the wide scope of areas covered. Brain Computer Interfaces are an inherently interdisciplinary field of research, and this book makes a point of covering in relative depth each of the fields involved. Researchers from a specific field of expertise will naturally benefit the most from the material covering areas outside of their own specialty. The subtitle, “Principles and Practise” clearly indicates the double purpose of this book: 

1. to present the scientific material necessary to understand the fundamental elements of a BCI system, its organization and its function;

2. to debate about the actual usage of Brain Computer Interfaces, especially with regard to a target population of disabled people.

Part II presents introductory notions about the brain and its activity, especially that which can be recorded as signals useful for BCI. Brain anatomy is reviewed, and the relation between different areas and brain function is established, in motor, visuomotor and somatosensory contexts. Focussing on motor‐related areas is a biased choice because other types of activity can also be used in BCI (visual, auditory, P300) ‐ neurophysiological aspects of these will be presented in later chapters, but in less detail. Chapter 2 also includes a discussion on the information conveyed by Action Potentials, and the means of measuring these within the brain, the technical details of which are somewhat overrepresented (also treated in chapters 5, 9, 16...). On the other hand, more detailed accounts of membrane depolarization, and action potential generation and propagation, could have been useful to find here.

Correlates of brain activity that can provide useful signals for BCI are either of the electromagnetic (chapter 3) or metabolic (chapter 4) kind. The neuronal origin of the electromagnetic fields of the brain is covered in chapter 3, along with considerations on their measurement, modeling, preprocessing and interpretation. Some of this material is redundant with chapters 6 (recording outside the brain) and 7 (feature extraction). Although biophysical aspects are treated in depth, the mathematical inverse source localization problem is described in a rather superficial manner. The advantage of exploiting electromagnetic fields of the brain for BCI is that the time scale at which these are modulated by brain behavior is very fast, typically tens of milliseconds. This makes for reactivity, which is a desirable feature in BCI. Other brain imaging modalities (fNIRS, fMRI), sensitive to oxygen consumption of brain tissue, vary according to time‐scales close to the second. Such *metabolic* imaging devices are presented in chapter 4. The topic of metabolic brain activity is also the topic of chapter 18, which presents pratical BCI applications. Although less relevant on its own than EEG for communication through a BCI on account of its slow dynamics, fNIRS could be combined with EEG, to bring in some additional information on the spatial localization of brain activity.

Part III contains seven chapters (chapters 5 to 11), concerned with BCI design, implementation and operation. Chapters 5 and 6 are about brain signal acquisition, from within the brain with microelectrode arrays (chapter 5) or outside the brain with EEG and MEG (chapter 6). These two chapters contain many extremely valuable practical considerations, such as the longevity of intracranial recordings, or the importance of the reference in EEG. One can wonder why the acquisition of ECoG recordings is deferred until Part IV (chapter 15), since it would have found a logical place in this part. Likewise, the chapter on acquisition outside of the brain only involves recordings of electromagnetic nature, and not fNIRS.

Feature extraction logically follows acquisition, in chapter 7. The material is self‐contained, making the reader for instance acquainted with the basics of Fourier analysis. More complex notions such as wavelets, or independent component analysis, are briefly sketched. This can constitute a useful primer to signal processing for readers with a clinical background. The same can be said about chapter 8, an introduction to feature translation, i.e. the procedure which transforms the features extracted in chapter 7 into control signals for BCI. Most of the commonly used methods (linear discriminant analysis, Naive Bayes, Support Vector Machines), are presented at a fast pace, without resorting to much mathematical formalism. This can convey some basic notions to the non‐specialized reader, who will have to look for more detailed material elsewhere. Because of the great diversity of methods and techniques presented, these two chapters could have benefited from some synthetic tables or diagrams, to orient the reader.The next two chapters of Part III deal with informatics‐related concepts: the hardware and software involved (chapter 9) as well as BCI operating protocols (chapter 10). This material is less academic than the preceding two chapters, and it is valuable to find it here very clearly exposed. Chapter 11 on BCI applications makes a principled transition towards the last two parts of the book: Part IV on “existing BCI” and Part V on “using BCI”.

In part IV, seven chapters review all of the different types of existing BCI: according either to the type of features (P300 in chapter 12, sensorimotor rhythms in chapter 13, SSVEP in chapter 14), to the type of recordings (ECoG in chapter 15, inside the motor cortex in chapter 16, inside the parietal and premotor cortices in chapter 17, metabolic signals in chapter 18). Each of these chapters provide very detailed accounts of either successes reached or shortcomings remaining to be overcome.

Part V, called “using BCI”, is mainly dedicated to translational studies. Chapter 19, centered on BCI users and their needs, provides useful reflections on the gap between patients’ wishes and the expectations on their needs held by BCI developers. A remark on cultural relativism could have been interesting, because hierarchies of needs certainly differ according to cultural habits and country of residence. Chapter 20, on clinical evaluation of BCI, comprises pragmatic advice for putting BCI into the hands of patients and their caregivers. Very useful for those interested in launching a start‐up company to distribute BCI systems, chapter 21 exposes the commercial and legal aspects of the matter, using boxed paragraphs to summarize the most important points (an excellent idea that other chapters could have taken example on). The sobering fact that BCI may be considered an *orphan technology* is not overlooked. Two following chapters deal with BCI applications, beyond improving the communication or self‐care of disabled patients. Chapter 22 describes preliminary studies on the improvement of brain function through BCI, in several areas (epilepsy, post‐stroke recovery, attention‐deficit disorders, pain management). Chapter 23 reviews possible applications of BCI research directed to the general population, for instance in augmented cognition, or videogames. The sixth and last chapter of this part on “Using BCI” is devoted to ethical issues; BCI researchers, on account of their variety of backgrounds, are not necessarily acquainted with such issues, and should read this chapter carefully to be aware of some deep ethical questions raised by their research.

The conclusive chapter is quite open‐ended, since BCI is still a budding technology, whose outcome will mostly unfold in the future. Some words of advice are provided to guide future research in the areas where the problems are considered the most crucial by the authors: signal acquisition hardware; validation and dissemination; and reliability.

In summary, the only ‐ very minor ‐ criticism I can make to this book is that the wealth of information it contains could have been slightly better organized. A reference book on Brain Computer Interfaces was lacking up to now, and this excellent one is bound to be extremely useful to the researchers working in this interdisciplinary field. Wolpaw and Wolpaw’s book holds its double promise of explaining where BCI comes from and where it is heading.

The book has a clear dedication to designing BCIs that actually serve the needs of the disabled population. At present, once BCIs have achieved good results within research laboratories, they can be tested on populations of patients, through clinical studies. Proof has been made by more than twenty independent studies that BCI can help certain patients (e.g. neuromuscular deficient ALS) to communicate. For instance, Wolpaw’s group reports 10‐week usage at home by 6 patients, and up to 3 years for one patient. Actual appropriation of the technique by patients will develop when the technology becomes affordable and robust, when it is recognized by the healthcare system as a viable technique, and when it becomes distributed by privately held companies. It is difficult to presume at present whether / when / where this will happen, but reading this book provides all the important guidelines for knowledgeably embarking upon the BCI adventure.

## Abbreviations

ALS: Amyotrophic lateral sclerosis; BCI: Brain computer interfaces; ECoG: Electrocorticography; EEG: Electroencephalography; fNIRS: functional near infrared spectroscopy; fMRI: functional magnetic resonance imaging; MEG: Magnetoencephalography; P300: a specific component of the EEG signal, occurring 300 ms after an event; SSVEP: Steady state visual evoked potential.

## Competing interests

The author declares that she has no competing interests.

